# Dynamics and Application of Modern, Smart, and Active Elements or Structures

**DOI:** 10.3390/ma15248852

**Published:** 2022-12-12

**Authors:** Rafal Rusinek

**Affiliations:** Department of Applied Mechanics, Mechanical Engineering Faculty, Lublin University of Technology, 20-618 Lublin, Poland; r.rusinek@pollub.pl

The Special Issue (SI) “Dynamics and Application of Modern, Smart, and Active Elements or Structures” is focused on covering all of the newest outcomes and trends in the nonlinear mechanics of systems and structures with smart, active, and modern materials. The modeling, machining, testing, and controlling of nonlinear dynamical systems is a key point of the SI. For this purpose, dozens of articles were submitted for publication in this SI, covering various aspects of the topic beginning from polymer and micro-fiber composites testing, carbon nanotube plates analysis, magneto-rheological dampers, and finishing on active implants for the middle ear. In total, 13 articles were published from scientists and researchers from all around the globe. A comparison of these articles with other modern articles on this topic has been carried out, which proves the relevance of this SI.

Smart materials (SMs) are defined as the materials that change their behavior in systematic manner as a response to specific stimulus (introduced by Rogers in 1989) which could be altered in magnetic and/or electric fields, stress, acoustic, temperature, nuclear radiation, and/or chemical properties. They are superior to other materials with five characteristics: selectivity, directness, immediacy, self-actuation, and transiency (by Addington and Schoedeck in 2006).

A smart structure is described as a system containing multi-functional parts that can perform sensing, control, and actuation. It can be understood as a primitive analogue of a biological body. SMs are used to construct these smart structures, which can perform both sensing and actuation functions. Generally, for actuators, four types of materials are used:

- Shape memory alloys: these are metals that, after having been deformed, revert to their original shapes when the temperature is changed;
- Piezoelectric ceramics: these expand and contract in response to an applied electrical field; conversely, they similarly generate an electrical field when their dimensions are altered;
- Magnetostrictive materials: they are analogous to that of the piezoelectric, except that they are responsive to changes in a magnetic field;
- Electrorheological/magnetorheological fluids: these are liquids that experience changes in viscosity upon the application of an electrical field and a magnetic field.

While, for sensors, usually are used:

- Optical fibers;
- Piezoelectric materials (e.g., polymers);
- Micro-electromechanical systems (MEMS).

All the smart sensors and actuators can find various applications in modern structures. What is more, it is difficult to find any modern device without an electronic sensor or actuator. Therefore, the problem of smart elements and structures is of key importance, as well as the problem of active elements (components). According to electronic device definition, an active components are those that deliver or produce energy or power in the form of a voltage or current. Passive components are those that utilize or store energy in the form of voltage or current. However, active elements have a bit of a different meaning in various field of technology, e.g., a prosthesis of the middle ear is called as passive implant, while an implantable middle ear hearing device is called an active implant.

Thus, this SI is a combination of different applications and analyses of smart and active structures in many different fields of science and technology. In this editorial, we present a short introduction of all articles published in this SI.

Mishra et al. [1] presented free vibration response of angle ply laminates with uncertainties using a Multivariate Adaptive Regression Spline (MARS), Artificial Neural Network-Particle Swarm Optimization (ANN-PSO), Gaussian Process Regression (GPR), and Adaptive Network Fuzzy Inference System (ANFIS). A collection of four-mode shapes were created and the comparison study was performed to compare these results with findings presented in the literature and they were found in good agreement.

Zagorski et al. [2] showed an analysis of the influence of technological parameters related to tool holder types on vibrations occurring during the milling of AZ91D magnesium alloy. The tool used in the study was a carbide end mill with TiAlN coating, clamped in three different types of tool holder: ER collet, heat shrink, and Tendo E hydraulic. The milling tests used straight toolpaths at varied cutting speeds and feed per tooth values. Based on the vibration displacement and acceleration signals recorded during the machining tests, the statistical analysis of the vibrations were made. As part of the study, composite multiscale entropy (CMSE) analysis was also performed, describing the level of disorderliness of the obtained vibration signals. It was noted that multiscale entropy might be an important parameter describing the vibration signal.

Rusinek et al. [3] investigated quasi-periodic and chaotic vibrations in the human middle ear stimulated by an implant, which is fixed to the incus by means of a nonlinear coupler. The coupler represented a classical element made of titanium and shape memory alloy. A five-degrees-of-freedom model of lumped parameters was used to represent the implanted middle ear for both normal and pathological ears. The model was engaged to find numerically the influence of the nonlinear coupler on stapes and implant dynamics. As a result, regions of parameters regarding the quasi-periodic, polyharmonic, and irregular motion were identified. The nonlinear coupler caused irregular motion, which is undesired for the middle ear. However, it was proved that the use of the stiff coupler also ensures regular vibrations of the stapes for higher frequencies. As a consequence, the utility of the nonlinear coupler was proven.

Ambrozkiewicz et al. [4] analyzed the energy efficiency of a Micro Fiber Composite piezoelectric system. It was based on a smart Lead Zirconate Titanate material that consists of a monolithic PZT (piezoelectric ceramic) wafer, which is a ceramic-based piezoelectric material. An experimental test rig consisting of a wind tunnel and a developed measurement system was used to conduct the experiment. The developed test rig allowed changing the air velocity around the tested bluff body and the frequency of forced vibrations, as well as recording the output voltage signal and linear acceleration of the tested object. The mechanical vibrations and the air flow were used to find the optimal performance of the piezoelectric energy harvesting system.

Li et al. [5] designed a variant truss beam structure with a large shrinkage ratio and high impact resistance. Based on the principle of the curved trajectory of scissor mechanisms, authors conducted a finite element simulation analysis of the impact load on the truss beam structure, a theoretical analysis of the impact response and a relevant prototype bench-top experiment, completing a full study on the impact resistance mechanism of the designed variant truss beam structure under the impact load. Moreover, the buffer effect of the external load impact on the variant truss beam structure was analyzed from the perspective of the energy change of elastic–plastic deformation. This paper proposes an optimization strategy for the variant truss beam structure with the energy absorption rate as the optimization index through extensive analysis of the parameter response surfaces. The strategy integrates analyses on the response characteristic analysis of various configuration materials to obtain an optimal combination of component parameters that ensures that the strength of the truss beam structure meets set requirements.

Kosicka et al. [6] determined the effect of a selected physical modifier with different granularity and mass percentage on the dynamics of aerospace polymer composites. The tests were carried out on samples made of certified aerospace materials used, among other purposes, for the manufacture of aircraft skin components. The hybrid composites were prepared from L285 resin, H286 hardener, GG 280T carbon fabric in twill 2/2, and alumina. The manufactured composites contained alumina with grain sizes of F220, F240, F280, F320, and F360. The mass proportion of the modifier in the tested samples was 5% and 15%. The tested specimens, as cantilever beams fixed unilaterally, were subjected to kinematic excitation with defined parameters of amplitude and frequency excitation in the basic resonance zone of the structure. The results, obtained as dynamic responses, were presented in the form of amplitude-frequency characteristics. These relationships clearly indicate the variable nature of composite materials due to modifier density and grain size.

Dabrowski et al. [7] presented the method of stability determination based on the value of the largest Lyapunov exponent (LLE). Since the stability problem is important also in smart structures, authors showed that LLE can be estimated from the vector field properties by means of the most basic mathematical operations. The article introduced new methods of LLE estimation for continuous systems and maps and showed that application of the approach can introduce significant improvement of the efficiency.

Gyliene et al. [8] suggested an improvement for stapes prosthesis stability. Numerical 3D models of a standard, as well as a modified (adjustable angled) stapes prosthesis were created in order to achieve this aim. Consequently, the modal analysis was performed to evaluate the mechanical behavior of the prosthesis, assuming that the piston would be made of Teflon, and the thin part, fixated on the incus long process, would be made from titanium alloy. Finally, the numerical analysis was conducted by changing the boundary conditions in respect of the prosthesis constraining. The modified stapes prosthesis, proposed in this research, allows avoidance of the negative effects of the over-constrained standard stapes prosthesis that appear over time. Moreover, the proposed modified prosthesis helps to regain hearing when the angle between the incus long process and prosthesis is unfavorable.

Zhang et al. [9] designed a frequency adjustable tuned mass damper (FATMD) based on a magneto rheological elastomer (MRE), which is a new type of magneto rheological smart material, with adjustable stiffness, obtained by changing the magnetic induction. Authors used MRE to change the stiffness of FATMD and to track the natural frequency of the main structure. However, adding TMD can change the natural frequency of the system. Therefore, it was used a combined Hilbert–Huang transform (HHT) and a natural excitation technique (NExT), with Simulink/dSPACE, to identify the natural frequency of the system in real time. Then, the natural frequency of the main structure through the TMD optimal design theory was calculated. This can help adjust FATMD to its optimum tuning state. To verify the applicability and effectiveness of FATMD, this paper compares the FATMD and traditional TMD experimental results. The experimental results indicate that FATMD, using the frequency tracking method, can effectively track the natural frequency of the main structure to ensure that the system is always in the optimum tuning state. In addition, FATMD can still achieve a good vibration reduction effect when the natural frequency of the main structure changes.

Kumar et al. [10] investigated the axial and shear buckling of a carbon nanotube (CNT)-reinforced multiscale functionally graded material (FGM) plate. Modified third-order deformation theory (MTSDT) with transverse displacement variation was used. CNT materials were assumed to be uniformly distributed, and ceramic fibers are graded according to a power-law distribution of the volume fraction of the constituents. The effective material properties were obtained using the Halpin–Tsai equation and Voigt rule of the mixture approach. A MATLAB code was developed using 9 noded iso-parametric elements containing 13 nodal unknowns at each node. The shear correction factor was eliminated in the present model, and top and bottom transverse shear stresses were imposed null to derive higher-order unknowns. Comparisons of the present results with those available in the literature confirm the accuracy of the existing model. The effects of material components, plate sizes, loading types, and boundary conditions on the critical buckling load were investigated. For the first time, the critical buckling loads of CNT-reinforced multiscale FGM rectangular plates with diverse boundary conditions were given.

Szmit et al. [11] did the experimental analysis of the dynamics and aerodynamic loads of a three-bladed rotor. The experimental tests focused on the rotation with three different angular velocities, for each angular speed, four different preset angles of beam were studied. During the laboratory experiment, strain gauges, as well as high-speed cameras, were used as the measurement system. The images from the high-speed cameras have been used to obtain aerodynamic loads in the form of polynomials, while the signals from strain gauges mounted on each beam allowed to observe the synchronization phenomenon between beams.

Wei et al. [12] studied the actuating performance of an micro fiber composite (MFC) in a sandwich structure, according to its action characteristics. The MFC was divided into upper and lower actuating units without any interaction between to two under the condition of plane strain, and the shear lag effect is considered between the units and the top and bottom of the sandwich structure. The actuating force of the MFC ends was obtained by considering its influence on the bending deformation of the sandwich structure, which deduces the actuating force formula of the embedded MFC. In contrast to ANSYS piezoelectric simulation, the distribution of the MFC interior normal stress is similar to the result from ANSYS piezoelectric simulation, and there is a very small deviation between the MFC end and central normal stress and the result from ANSYS piezoelectric simulation. Taking the end deflection of the sandwich structure with an embedded MFC as an example, the actuating force simulation of the MFC considering the shear lag effect is compared with the ANSYS piezoelectric simulation and actuating force simulation based on the Bernoulli–Euler model. The result indicates that the actuating force simulation of the MFC considering the shear lag effect is closer to the ANSYS piezoelectric simulation, which proves the rationality and necessity of considering the shear lag effect and end actuating force of the MFC.

Ciecielag et al. [13] presented results of ultrasonic non-destructive testing of carbon fibre-reinforced plastics (CFRPs) and glass-fibre reinforced plastics (GFRPs). First, ultrasonic C-scan analysis was used to detect real defects inside the composite materials. Next, the composite materials were subjected to drilling in the area of defect formation, and measured forces were used to analyze the drilling process using recurrence methods. Results have confirmed that recurrence methods can be used to detect defects formed inside a composite material during machining.

In conclusion, the editor wishes to give a special thanks to all the authors and the editorial team of *Materials* for the collaborative peer-review process. As for the readers, I hope you enjoy reading this Special Issue “Dynamics and Application of Modern, Smart and Active Elements or Structures” and discover new innovations to inspire future research.
